# Metal Nanoclusters/Polyvinyl Alcohol Composite Films as the Alternatives for Fabricating Remote-Type White Light-Emitting Diodes

**DOI:** 10.3390/nano12020204

**Published:** 2022-01-08

**Authors:** Zhaoyu Liu, Dong Yao, Huiwen Liu, Hao Zhang

**Affiliations:** 1 State Key Laboratory of Supramolecular Structure and Materials, College of Chemistry, Jilin University, Changchun 130012, China; zhaoyul18@mails.jlu.edu.cn (Z.L.); liuhuiwenjlu@163.com (H.L.); 2 Joint Laboratory of Opto-Functional Theranostics in Medicine and Chemistry, The First Hospital of Jilin University, Changchun 130021, China; 3 Green Catalysis Center, College of Chemistry, Zhengzhou University, Zhengzhou 450001, China

**Keywords:** metal nanoclusters, fluorescence, polyvinyl alcohol, composite film, light-emitting diodes

## Abstract

Packing luminescent metal nanoclusters (MNCs) into polymers and fabricating novel MNCs/polymer composite materials is effective in obtaining high-performance light-emitting diodes (LEDs). Herein, water soluble Cu and Au nanoclusters are encapsulated in polyvinyl alcohol (PVA) by a casting method. The obtained MNCs/PVA composite films are highly emissive with triple primary colors, and inherit the merits of PVA, such as transparency, flexibility, machinability, stability and self-healing ability. By employing the MNCs/PVA composite films as down-conversions, remote type monochromic and white LEDs are fabricated. The white LEDs (WLEDs) exhibit a maximum color rendering index (CRI) of 86 with a Commission Internationale de l’Eclairage (CIE) color coordinate of (0.33,0.35). By varying the three MNCs/PVA film arrangement, the correlated color temperature (CCT) of the WLEDs is tuned from 5582 to 9490 K, which signifies the possibility of MNCs/PVA as alternative light-emitting materials for advanced illumination and display in the future.

## 1. Introduction

Light-emitting nanomaterials, such as rare earth phosphors, colloidal quantum dots, and perovskites, embedder in resins, have been widely used as down-conversion layers in light-emitting diodes (LEDs) for illumination and display [1,2,3,4,5,6]. Despite the excellent performances in optical properties, these light-emitting materials still suffers from tedious production, high price and/or high toxicity [7,8,9]. To meet the requirements of “green development” concept, novel nontoxic, low cost and easily available light-emitting materials are greatly welcome [10,11,12].

Metal nanoclusters (MNCs), representatively, Au, Ag and Cu NCs, are one of the ideal candidate lighting materials [13,14,15,16,17]. Composed of several to hundreds of metal atoms, these MNCs possess sizes close to the Fermi wavelength of electrons and exhibit molecular-like optical properties, such as discrete energy levels and strong and tunable photoluminescence (PL) [18,19,20,21]. Additionally, what is even more important is that these MNCs are heavy-metal-free and can be easily obtained. Even so, MNC-based LEDs still have two drawbacks: (i) MNCs possess less PL stability than traditional light-emitting materials, especially when exposed to the stimulus such as heat, oxygen and moisture [22]; (ii) the conventional LED structure is to coat the light-emitting materials embedded in resins onto the UV chips. The direct contact to the UV chips results in continuous heat, and further leads to the phase separation, color inhomogeneity and efficiency deterioration [23,24,25,26,27]. Packaging the MNCs into polymers offers the possibility to overcome the above-mentioned shortcomings [28,29,30].

Polyvinyl alcohol (PVA) is a commercialized polymer, which has been widely used as polarizer in the liquid crystal imaging system and approved by the USA Food and Drug Administration (FDA) for medical applications [31,32,33,34]. Apart from low cost and high biosafety, PVA also exhibits other advantages, such as compatibility, transparency, recyclability and ease of processing, which makes it a preferred host matrix for fabricating MNCs/polymer composite materials [35,36]. Packaging MNCs into MNCs/PVA composite materials, on the one hand, could protect MNCs from the external environmental stimulus, which improves the stability of the LED. On the other hand, the free-standing nature of the composite material make it possible to avoid the direct contact between MNCs and UV chips and build a remote-type structure, which could promote the light output performance of the LEDs [22].

In this work, 2-mercapto-1-methylimidazole capped Cu nanoclusters (Cu-MMI NCs), 6-Aza-2-thiothymidine capped Au nanoclusters (Au-ATT NCs) and bovine serum albumin capped Au nanoclusters (Au-BSA NCs) are cast into MNCs/PVA composite films with bright blue, green, and red emission, respectively. The MNCs/PVA composite films not only inherit the emissive property of the MNCs, but also possess the merits of high transparency, flexibility, stability and self-healing ability from PVA. As a proof of concept, the MNCs/PVA composite films are further employed as down-converters to build remote-type monochromic and white LEDs, which exhibit a maximum color rendering index (CRI) of 86 and correlated color temperature (CCT) ranging from 5582 to 9490 K.

## 2. Materials and Methods

### 2.1. Materials

Copper chloride dihydrate (CuCl_2_·2H_2_O, 99.99%), 2-mercapto-1-methylimidazole (MMI, 98.0%), L-arginine (Arg, 99.0%) and bovine serum albumin (BSA, 98.0%) were purchased from Aladdin Reagent Company, Shanghai, China. 6-Aza-2-thiothymidine (ATT) was purchased from Alfa Aesar Chemical Co., Ltd., Shanghai, China. Chloroauric acid (HAuCl_4_·4H_2_O, Au mol% > 47.8%) and sodium hydroxide (NaOH) were purchased from Sinopharm Chemical Reagent Co., Ltd., Shanghai, China. Polyvinyl alcohol 1788 (PVA) was purchased from Chengdu Kelong Chemical Co., Ltd., Chengdu, China. All the materials were used as received without further purification.

### 2.2. Preparation of Cu-MMI NCs

Cu-MMI NCs were synthesized according to the previous report with slight modification. In summary, 14.0 mg of CuCl_2_·2H_2_O was dissolved in 5 mL of water and stirred until completely dissolved. Subsequently, 37.7 mg of MMI and 118.8 mg of NaOH were added under vigorous stirring. The reaction was maintained at 55 °C in the water bath for 50 min, and the obtained crude product was purified with centrifugation.

### 2.3. Preparation of Au-ATT NCs

Au-ATT NCs were synthesized according to the previous report with slight modification. In summary, 5 mL of HAuCl_4_·4H_2_O (10 mmol/L) and 5 mL of ATT (80 mmol/L) were mixed and stirred for 2 min. Subsequently, 0.05 mol of NaOH solution was added to adjust the pH to weakly alkaline. After stirring in the dark at room temperature for 1 h, the obtained crude product was purified by dialysis. Then, 1 mL of Arg (40 mmol/L) was added to the as-prepared solution and the pH was adjusted to 10. The solution was stirred at 37 °C for 24 h until the Au-ATT NCs with bright green emission was obtained.

### 2.4. Preparation of Au-BSA NCs

To synthesize Au-BSA NCs, 5 mL of HAuCl_4_·4H_2_O (10 mmol/L) and 5 mL of BSA (50 mg/mL) were mixed and stirred for 2 min. Subsequently, 0.5 mL of NaOH (1 mol/L) was added to adjust the pH to weakly alkaline. The reaction was maintained in a water bath at 37 °C for 24 h, and the obtained crude product was purified by dialysis.

### 2.5. Preparation of MNCs/PVA Composite Film

Totals of 0.5 mL of the as-prepared MNC aqueous solution (5~30 mg/mL) and 1.5 mL of the PVA aqueous solution (mass fraction 5~12%) were mixed at room temperature and stirred for 30 min. Subsequently, the mixture was centrifuged at 2000 rpm for 3 min to remove air bubbles. Then, the mixture was evenly dropped onto a cleaned glass slice and cured at room temperature.

### 2.6. Characterization

Transmission electron microscopy (TEM) was recorded on a JEM-2100F electron microscope (Hitachi, Tokyo, Japan). The dynamic light scattering was measured by a Zetasizer NanoZS (Malvern, Shanghai, China). UV-visible absorption spectra were recorded using a Shimadzu 3100 UV-vis spectrophotometer (Shimadzu, Tokyo, Japan). Fourier-transform infrared (FTIR) spectroscopy were performed with a VERTEX 80V FTIR instrument (Bruke, Karlsruhe, Germany). Photoluminescence spectroscopy was carried out on a Shimadzu RF-5301 PC spectrophotometer (Shimadzu, Tokyo, Japan). X-ray powder diffraction (XRD) investigation was performed on an Empyrean X-ray diffractometer (Malvern, Shanghai, China) using Cu K radiation (λ = 1.5418 Å). Atomic force microscope (AFM) tapping mode measurements were performed on a Bruke scanning probe microscope (Bruke, Madison, WI, USA) using a rotated tapping mode etched silicon probe tip. SEM micrographs were measured by JEOL-FESEM 6700F electron microscope (JEOL, Tokyo, Japan) with a primary electron energy of 3 kV. Before imaging, the samples were sputter-coated with 2 nm Pt. The luminescence decay curves were measured on Edinburgh FLS920 (Techcomp, Shanghai, China) equipped with an integrating sphere (excited at 365 nm). An FESEM 6700F electron microscope (JEOL, Tokyo, Japan) was used to observe the process of self-healing. The spectra of the LEDs were measured at room temperature in the open air with a Spectrascan PR-650 spectrophotometer (JADAK, New York, NY, USA) equipped with an integrating sphere. The CIE (Commission Internationale de L’Eclairage 1931) calorimeter system was used to identify the color of the light.

## 3. Results and Discussion

### 3.1. Preparation and Optical Properties of MNCs/PVA Solutions

To obtain the MNCs/PVA solutions, blue-emissive Cu-MMI NCs, green emissive Au-ATT and red-emissive Au-BSA NCs were foremost synthesized according to previous reports [37,38,39]. All the MNCs are well dispersed with uniform size distribution (Figure S1). The average diameter of Cu-MMI, Au-ATT and Au-BSA NCs is 2.1, 2.6 and 2.4 nm, respectively. Desired amount of MNCs were then blended with PVA solutions to form homogenous MNCs/PVA solutions, where the MNCs mass fraction is 2.5%. Figure S2 illustrates the absorption and PL emission of the MNCs dispersed in aqueous solution and PVA solution. The photoluminescence emission quantum yields (PLQYs) of both the MNCs are measured to be 6.4, 2.5 and 5.3%, respectively. The MNCs/PVA composite solutions retain the optical properties of the MNCs. Only the spectra of Au-ATT NCs/PVA solution and Au-BSA NCs/PVA solution show a red shift of a few nanometers compared with those of the Au-ATT NCs and Au-BSA NCs aqueous solution, which could be attributed to interactions between the MNCs and PVA molecules and the electronic environment variation of the MNCs (Table S1). As shown in Figure S3 and Table S2, the PL lifetime of the MNCs/PVA solutions is significantly improved compared with the MNCs aqueous solutions. On the incorporation of PVA, due to the interactions, the vibration, rotation and torsion motion of the MNCs are restricted, which reduces the possibility of non-radiation paths and enhances the radiation attenuation, thereby extending the PL lifetime.

### 3.2. Structural and Optical Characterization of the MNCs/PVA Composite Films

**Figure 1:**
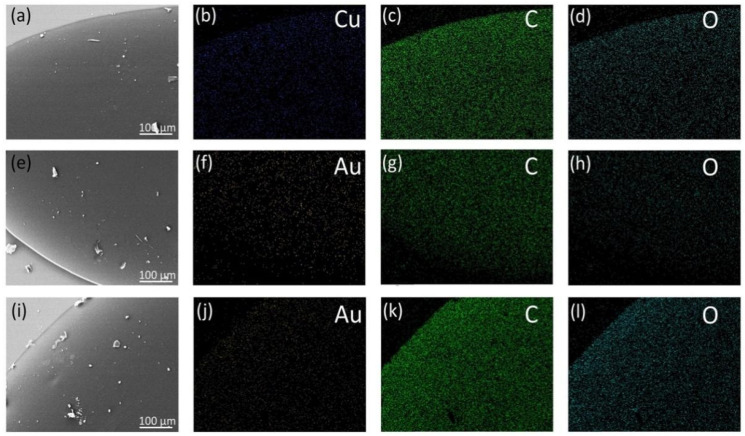
(**a**) SEM image and (**b**–**d**) SEM-EDS elemental mapping of Cu-MMI/PVA film, (**e**) SEM image and (**f**–**h**) SEM-EDS elemental mapping of Au-ATT/PVA film, and (**i**) SEM image and (**j**–**l**) SEM-EDS elemental mapping of Au-BSA/PVA film.

MNCs/PVA solutions were dehydrated into films by a casting method. The obtained composite films are freestanding and can be easily peeled off from the substrate. The scanning electron microscopy (SEM) equipped with energy-dispersive X-ray spectroscopy (EDS) reveals that all the MNCs/PVA composite films are composed of three main elements, Cu/Au, C and O and the MNCs are homogenously dispersed in the PVA matrix (Figure 1). The average roughness of the Cu-MMI NCs/PVA, Au-BSA NCs/PVA and Au-ATT NCs/PVA films is 2.57, 1.86 and 1.11 nm, respectively, implying the smooth surface of the composite films (Figure S4). The roughness of the Cu-MMI NCs/PVA film is a little higher than those of the Au-BSA NCs/PVA and Au-ATT NCs/PVA films, which can be ascribed to the higher degree of aggregation of Cu-MMI NCs in PVA [40,41,42].

To explore the interaction between MNCs and PVA in the composite films, X-ray radiation diffraction (XRD) is performed. For the pure PVA film, there is a broad diffraction peak centered at 19.3 degrees, corresponding to the spacing of (101), which reflects the semi-crystalline characteristics of PVA (Figure S5). The semi-crystalline nature endows PVA with a mass of free voids between the polymeric chains in the amorphous phase, where the MNCs could become trapped. Compared with pure PVA, the XRD pattern of MNCs/PVA composites show no appearance of new crystalline peak, but slight red shifts, revealing that the MNCs are well-dispersed in PVA, and does not change the basic structural configuration of PVA. The distance decrement of the adjacent PVA molecules is ascribed to the interactions between the MNCs and PVA, probably with the hydroxyl groups [43,44]. The weak bonding effect in the MNCs/PVA composite is also affirmed by the FTIR analysis. The IR spectra of the MNCs/PVA composite films maintain the fundamental vibrational bands of PVA without the appearance of any additional peak (Figure S6). Comparatively, the characteristic stretching vibration peak of the hydroxyl group in PVA at 2947 cm^−1^ shifts to 2942 in Cu-MMI NCs/PVA, 2942 cm^−1^ in Au-ATT NCs/PVA and 2938 cm^−1^ in Au-BSA NCs/PVA, respectively [45]. These results indicate that no covalent bonds form between the MNCs and PVA and the incorporation of MNCs hardly affects the basic PVA structure, which is consistent with the XRD result. 

**Figure 2:**
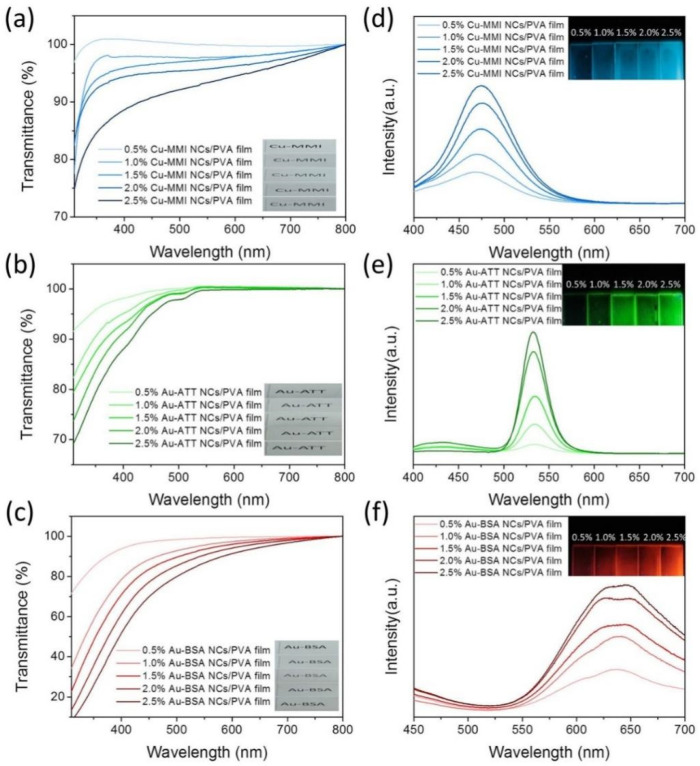
The transmission spectra of (**a**) Cu-MMI NCs/PVA film, (**b**) Au-ATT NCs/PVA film, and (**c**) Au-BSA NCs/PVA film with different NC concentrations. Insets of (**a**–**c**) exhibit the transparency of the composite films with corresponding photographs under room-light. The PL spectra of (**d**) Cu-MMI NCs/PVA film, (**e**) Au-ATT NCs/PVA film, and (**f**) Au-BSA NCs/PVA film with different NC concentrations. Insets are the corresponding photographs under excitation of 365 nm.

The MNCs/PVA composite films are so transparent that printed letters beneath the MNCs/PVA films can be clearly seen (Figure 2a–c). With a 0.5% mass fraction of MNCs, the transmission of the three MNCs/PVA exceeds 70% in both the ultraviolet and visible range. When the MNC mass fraction is increased up to 2.5%, Cu-MMI NCs/PVA films and Au-ATT NCs/PVA films achieve a transmission over 80% in the entire visible range, whereas the transmission of the Au-BSA NCs/PVA film remains over 70% in the range of 450 to 900 nm (Figure 2a–c). The minor absorption of visible light is beneficial for the fabrication of light-emitting diodes (LEDs). With the increment of the MNCs mass content, the PL intensity of the three MNCs/PVA composite films increase linearly, accompanied by the slight red shift of the PL peak position, which originates from the growing degree of MNCs aggregation (Figure 2d–f, Figures S1 and S7). The PLQYs of the MNCs/PVA films are 11.4, 10.4 and 9.9%, and the average PL lifetimes are 10.7, 11.2 and 11.8 μs, respectively, which are significantly higher and longer than those of the corresponding MNCs (Figure S8). Based on the PLQYs and PL lifetimes, the radiative rate constant (k_r_) and nonradiative rate constant (k_nr_) of the MNCs and MNCs/PVA films are calculated (Table S3). After packing into the PVA films, the k_r_ of MNCs is obviously promoted, whereas the k_nr_ is inhibited. These phenomena are ascribed to the further vibration and rotation motion restriction of the MNCs in the PVA films.

### 3.3. PL Stability of MNCs/PVA Composite Films

**Figure 3:**
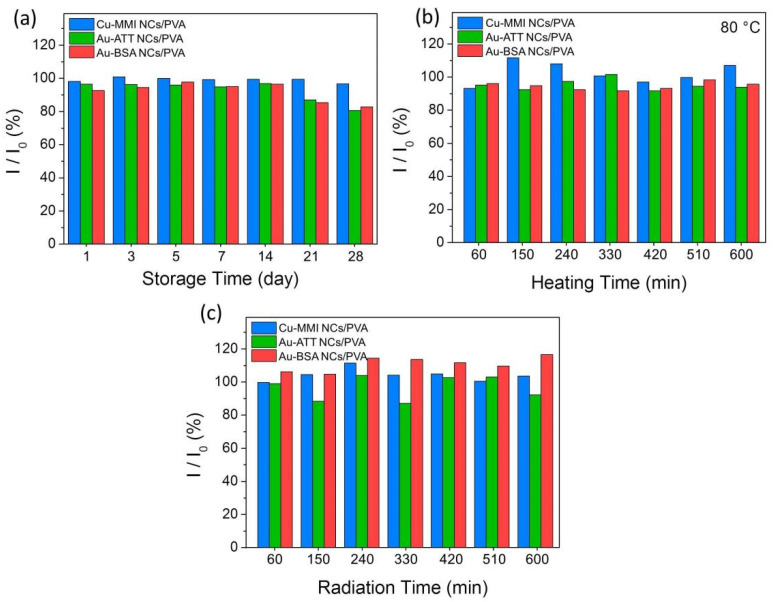
Stability of the Cu-MMI NCs/PVA film, Au-ATT NCs/PVA film, and Au-BSA NCs/PVA film: (**a**) storage stability, (**b**) thermal stability and (**c**) UV-irradiation stability.

PL stability is an important parameter for the practical application of photovoltaic and optoelectronic devices. Generally, MNCs are sensitive to environmental stimulus, which need to be stored in the dark at 4 °C. Encapsulating MNCs into polymers can effectively improve the PL stability of MNCs to the environment. In Figure 3a, the MNCs/PVA composite films exhibit negligible PL intensity loss after stored in an open environment (28 °C, humidity = 19%) for 14 days, and less than 20% PL intensity loss is recorded when the MNCs/PVA composite films are stored for 28 days, showing their good stability under ambient conditions. The MNCs/PVA composite films are heated to 80 °C in air to explore the thermal stability (Figure 3b). After heating for 10 h, more than 90% of PL intensity of the Au-ATT NCs/PVA and Au-BSA NCs/PVA composite films are preserved and the PL intensity of the Cu-MMI NCs/PVA composite film is even improved beyond the initial intensity. As shown in Figure 3c, after irradiation under 365 nm for 10 h, only minor changes in PL intensity of the MNCs/PVA composite films are recorded and more than 90% of PL intensity is preserved. Such good stability is attributed to the protection of PVA encapsulation to isolate the MNCs from the external environment, which is beneficial for the use of MNCs/PVA composite films in LEDs.

### 3.4. Fabricability and Self-Healing Ability of the MNCs/PVA Composite Films

**Figure 4:**
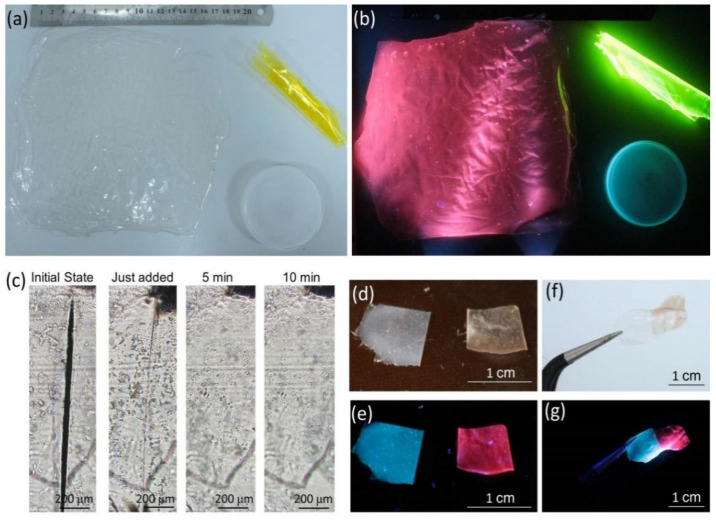
Photographs of the Cu-MMI NCs/PVA film, Au-ATT NCs/PVA film, and Au-BSA NCs/PVA film under (**a**) room light and (**b**) 365 nm excitation. (**c**) The light microscope photographs demonstrating the self-healing process of the Cu-MMI NCs/PVA film (from left to right). (**d**) The photographs of the separated Cu-MMI NCs/PVA and Au-BSA NCs/PVA films under room light and (**e**) under 365 nm excitation. (**f**) The photographs of the separated Cu-MMI NCs/PVA and Au-BSA NCs/PVA films under room light and (**g**) under 365 nm excitation.

Due to the flexible and stretchable nature of PVA, it is easy for the MNCs/PVA composite films to be cast into any shapes with specific sizes. As shown in Figure 4a, a large square Au-BSA NCs/PVA film with a size of 16 cm × 16 cm, a coil of Au-ATT NCs/PVA composite film and a disk of Cu-MMI NCs/PVA composite film are fabricated. The three shaped MNCs/PVA composite films emit bright red, green and blue light, respectively, preserving their initial emission properties (Figure 4b).

Moreover, the MNCs/PVA films also inherit the self-healing properties of PVA. As illustrated in Figure 4c, to repair the 10 μm wide incision on the Cu-MMI NCs/PVA film, 1 μL of water is dropped onto the incision. The incision narrows immediately and completely disappears in 5 min without external heating. The addition of the trace water promotes the intermolecular hydrogen bonding between adjacent PVA molecules and thus inducing the self-healing process. Based on the self-healing ability, two separated slices of 1 cm × 1 cm Cu-MMI NCs/PVA film and Au-BSA NCs/PVA film are assembled through side-by-side arrangement and assistance of water, demonstrating the possibility of the MNCs/PVA films to build multicolor pixel arrays (Figure 4d–g) [46].

### 3.5. Application of MNCs/PVA Composite Films in Remote-Type LEDs

**Figure 5:**
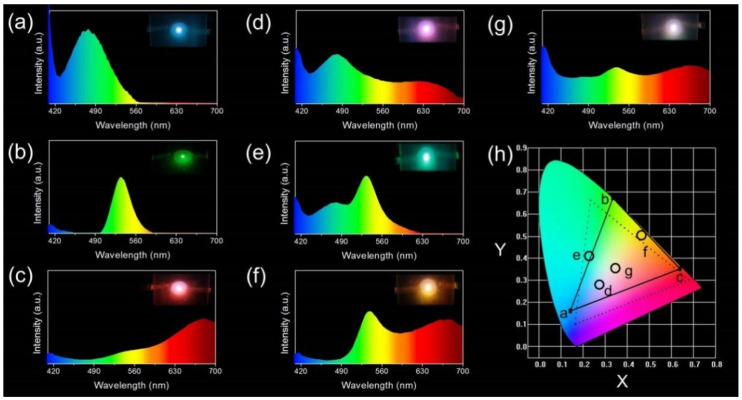
The emission spectra of the remote LEDs based on monolayer and multilayer MNCs/PVA films: (**a**) Cu-MMI/PVA film, (**b**) Au-ATT/PVA film, (**c**) Au-BSA/PVA film, (**d**) Cu-MMI/PVA film and Au-BSA/PVA film, (**e**) Cu-MMI/PVA film and Au-ATT/PVA film, (**f**) Au-ATT/PVA film and Au-BSA/PVA film and (**g**) Cu-MMI/PVA film, Au-ATT/PVA film, and Au-BSA/PVA film. Insets of (**a**−**g**) are the corresponding photographs of the remote LEDs. (**h**) The CIE chromaticity coordinates of the LEDs in (**a**–**g**). The dashed line is the range of color gamut of NTSC and the solid line is the color coverage of the LED based on our MNCs/PVA films.

Based on the properties discussed above, the MNCs/PVA composite films are considered to be excellent down-converter alternatives for LED application. Since the MNCs/PVA composite films are free standing, a remote-type LED architecture is applicable to avoid the direct contacts between the down-converters and the GaN chip, minimize the operating temperature and reduce phase segregation between MNCs and PVA, thus improving the LED performance (Figure S9). As shown in Figure 5a–c, a single layer of the MNCs/PVA film is employed to fabricate monochromic LEDs. The emission peaks of the blue, green and red LEDs are centered at 478 nm, 534 nm and 665 nm, respectively (Figure 5a–c). The corresponding CIE color coordinates of the three LEDs are (0.13, 0.16), (0.31, 0.67) and (0.62, 0.34), composing an RGB triangle which covers 80.1% of the NTSC color space (Figure 5h). To extend the emission color of the LED, a double layer of the MNCs is employed. As shown in Figure 5d–f, pink, turquoise and orange LEDs are obtained with the combination of Cu-MMI NCs/PVA film and Au-BSA NCs/PVA film, Cu-MMI NCs/PVA film and Au-ATT NCs/PVA film, and Au-ATT NCs/PVA film and Au-BSA NCs/PVA film, respectively, with corresponding chromaticity coordinates at (0.27, 0.29), (0.21, 0.40) and (0.44, 0.50) (Figure 5h).

To fabricate the WLED, triple-layer films are utilized together as the down-converter. The three composite films are arranged with a R-G-B sequence from bottom up, where R represents red-emissive Au-BSA NCs/PVA film, G represents green-emissive Au-ATT NCs/PVA film and B represents blue-emissive Cu-MMI NCs/PVA film. Figure 5g exhibits the photograph and spectrum of the WLED. The chromaticity coordinate of the WLED is (0.33, 0.35), which is close to the pure white light coordinate (0.33, 0.33). The color rendering index (CRI) and correlated color temperature (CCT) of the WLED is 86 and 5582 K, respectively, with a luminance of 89.36 cd/m^2^. Note that the low efficiency is mainly due to the poor quality of the GaN chip. As is known to all, reabsorption exists between different emitters with absorption and emission overlaps. Based on the reabsorption effect, various composite film arrangements are attempted to tune the property of the WLED (Figure S10). Apart from the R-G-B sequence, other five combinations based on the three MNCs/PVA films are list in Table S4. The emission of the WLEDs is tuned from pure white to cold white, with CCT ranging from 5582 to 9490 K (Table S4).

## 4. Conclusions

In summary, we succeeded in fabricating free-standing Cu-MMI NCs/PVA, Au-ATT NCs/PVA, Au-BSA NCs/PVA composite films by a simple casting method. The MNCs/PVA composite films are transparent and can emit three primary colors under 365 nm irradiation, respectively. Moreover, the MNCs/PVA composite films are easily processed into desired shapes and sizes, and exhibit excellent stability and self-healing ability. A remote-type WLED prototype with CIE coordinate of (0.33, 0.35) and high CRI of 86 is further fabricated by employing the MNCs/PVA composite films as down-converters, the CCT of which could be tuned through various MNCs/PVA film arrangements. Due to the nontoxicity and ease of processing, this work may promote the practical application of the luminescent MNCs/PVA composite films as down-converter alternatives for lighting.

## Supplementary Materials

The following are available online at https://www.mdpi.com/article/10.3390/nano12020204/s1, Figure S1: High magnification TEM images of (a) Cu-MMI NCs, (b) Au-ATT NCs and (c) Au-BSA NCs; Figure S2: The absorption spectra of (a) Cu-MMI NC and Cu-MMI/PVA solution, (b) Au-ATT NC and Au-ATT/PVA solution, and (c) Au-BSA NC and Au-BSA/PVA solution. (d–f) The PL spectra of corresponding MNCs and MNCs/PVA solution; Table S1: The position of PL emission peaks of MNCs, MNCs/PVA composite solutions and MNCs/PVA composite films; Figure S3: PL decay curves of the MNCs, MNCs/PVA composite solutions and films, which are detected under 365 nm excitation. (a) Cu-MMI NCs, (b) Au-ATT NCs, and (c) Au-BSA NCs; Table S2: The PL lifetime of the MNCs and MNCs/PVA composite solutions detected at 365 nm; Figure S4: 3D AFM images of the (a) Cu-MMI NCs/PVA film, (b) Au-ATT NCs/PVA film and (c) Au-BSA NCs/PVA film; Figure S5: XRD patterns of PVA film, Cu-MMI NCs/PVA film, Au-ATT NCs/PVA film, and Au-BSA NCs/PVA film; Figure S6: FTIR spectra of PVA, Cu-MMI NCs/PVA, Au-ATT NCs/PVA, and Au-BSA NCs/PVA composite films; Figure S7: The linear regression curves of the PL emission intensity of (a) Cu-MMI NCs/PVA, (b) Au-ATT NCs/PVA, and (c) Au-BSA NCs/PVA composite films versus the weight content of MNCs; Figure S8: PL decay curves of the MNCs/PVA composite films, which are detected under 365 nm excitation. (a) Cu-MMI NCs, (b) Au-ATT NCs, and (c) Au-BSA NCs. Table S3: Photophysical data of MNCs and MNCs/PVA composite films. Figure S9: The structure illustration of a remote-type LED; Figure S10: PL emission spectra of WLEDs based on the triple composite films with different arrangement of Cu-MMI NCs/PVA, Au-ATT NCs/PVA, and Au-BSA NCs/PVA films, where B represents blue, G represents green and R represents red. From bottom up: (a) B-G-R, (b) B-R-G, (c) G-R-B, (d) G-B-R and (e) R-B-G; Table S4: Index parameters of WLEDs based on the triple composite films with different arrangement of Cu-MMI NCs/PVA, Au-ATT NCs/PVA, and Au-BSA NCs/PVA films from the bottom up.
