# Supplementary material for: Metal Nanoclusters/Polyvinyl Alcohol Composite Films as the Alternatives for Fabricating Remote-Type White Light-Emitting Diodes
Source: Nanomaterials (Basel). 2022 Jan 8;12(2):204. doi: 10.3390/nano12020204 (PMC8778349; doi:10.3390/nano12020204)
Supplement: Supplementary file 1 [file nanomaterials-12-00204-s001.zip › nanomaterials-1490374-supplementary.pdf]

Supplementary Materials

Metal Nanoclusters/Polyvinyl Alcohol Composite Films as the Alternatives for Fabricating Remote-Type White Light-Emitting Diodes

Zhaoyu Liu [1], Dong Yao [1,*], Huiwen Liu [1,2] and Hao Zhang [1,3,*]

[1] State Key Laboratory of Supramolecular Structure and Materials, College of Chemistry, Jilin University, Changchun 130012, China; zhaoyul18@mails.jlu.edu.cn (Z.L.), liuhuiwenjlu@163.com (H.L.)
[2] Joint Laboratory of Opto-Functional Theranostics in Medicine and Chemistry, The First Hospital of Jilin University, Changchun 130021, China
[3] Green Catalysis Center, College of Chemistry, Zhengzhou University, Zhengzhou 450001, China
* Correspondence: dongyao@jlu.edu.cn (D.Y.); hao_zhang@jlu.edu.cn (H.Z.)

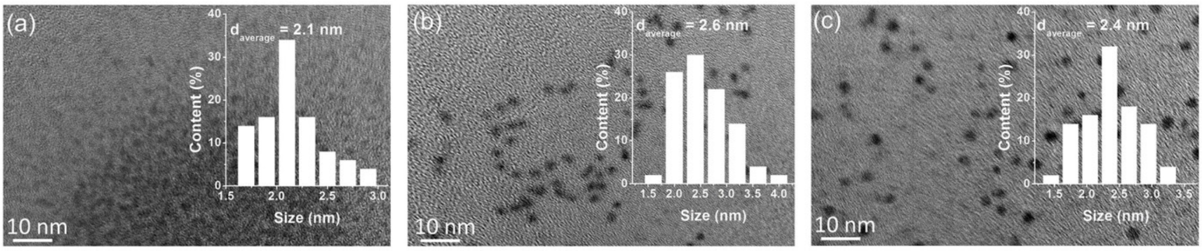

Figure S1. High magnification TEM images of (a) Cu-MMI NCs, (b) Au-ATT NCs and (c) Au-BSA NCs. Insets: the corresponding size distribution of the MNCs.

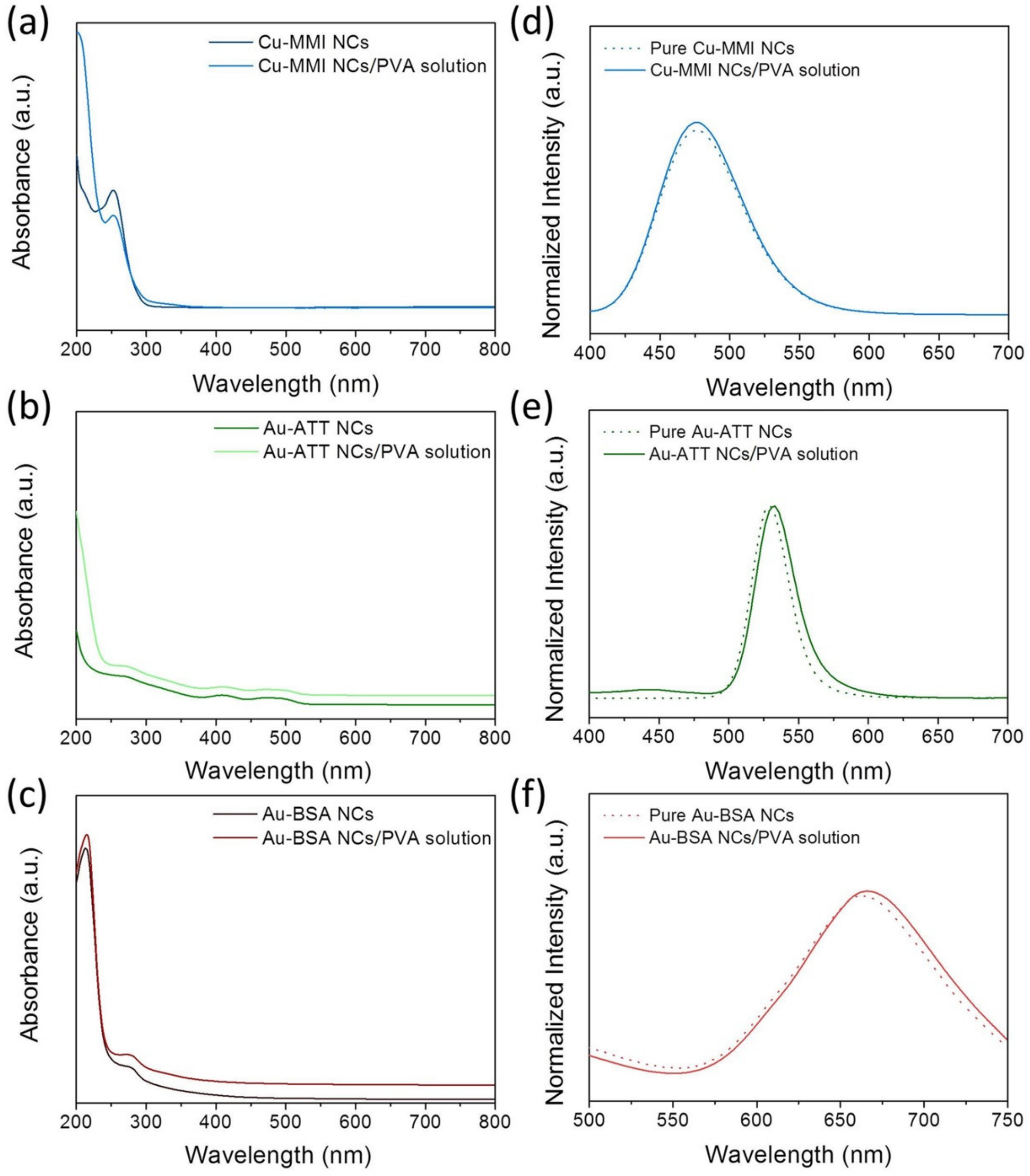

Figure S2. The absorption spectra of (a) Cu-MMI NC and Cu-MMI/PVA solution, (b) Au-ATT NC and Au-ATT/PVA solution, and (c) Au-BSA NC and Au-BSA/PVA solution. (d-f) The PL spectra of corresponding MNCs and MNCs/PVA solution.

Table S1. The position of PL emission peaks of MNCs, MNCs/PVA composite solutions and MNCs/PVA composite films.

	PL peak of MNCs (nm)	PL peak of MNCs/PVA Solution (nm)	PL peak of MNCs/PVA Film (nm)
Cu-MMI NCs	476	476	478
Au-ATT NCs	528	532	534
Au-BSA NCs	663	666	672

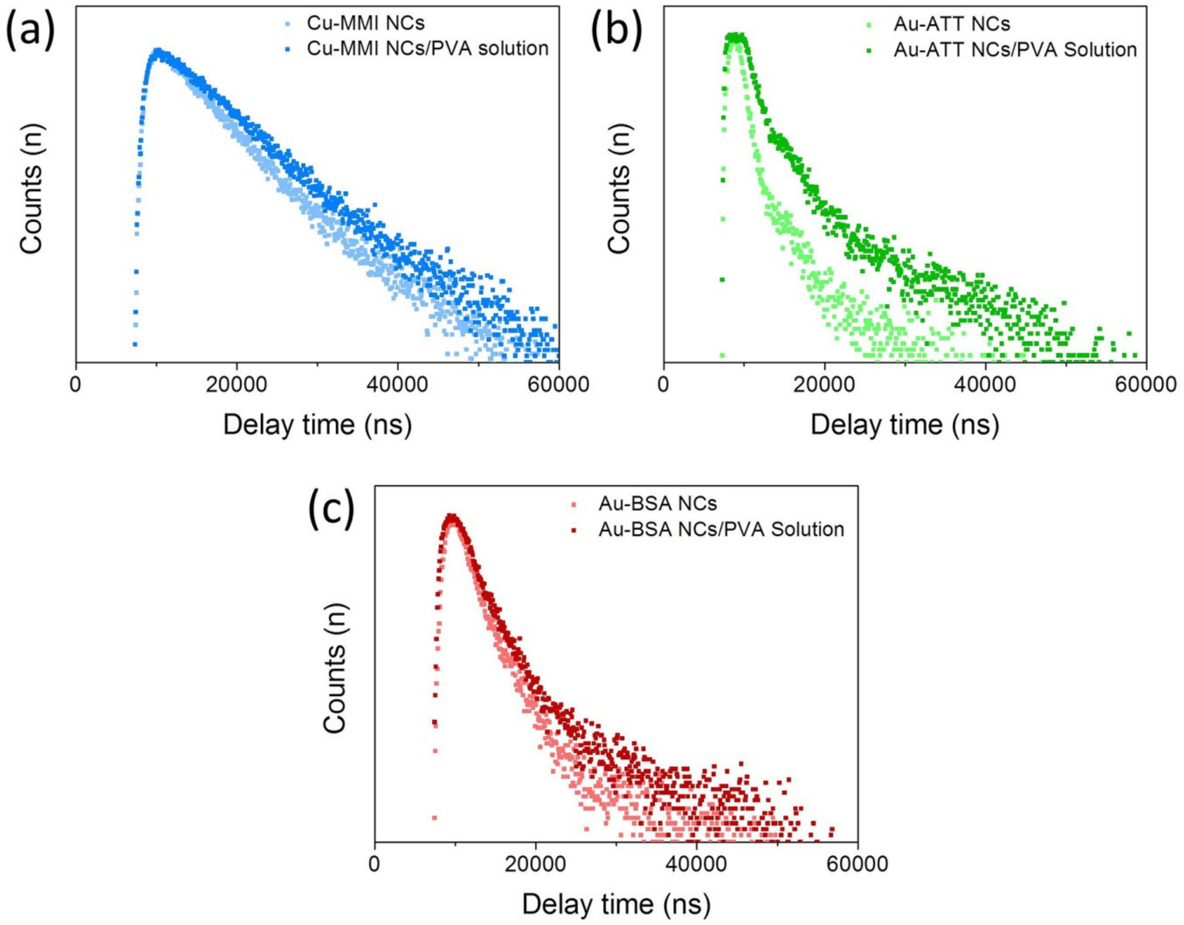

Figure S3. PL decay curves of the MNCs, MNCs/PVA composite solutions, which are detected under 365 nm excitation. (a) Cu-MMI NCs, (b) Au-ATT NCs, and (c) Au-BSA NCs.

Table S2. The PL lifetime of the MNCs and MNCs/PVA composite solutions detected at 365 nm.

	τ_1 (μs)	Rel$_{\tau 1}$ (%)	τ_2 (μs)	Rel$_{\tau 2}$ (%)	$\tau_{average}$ (μs)
Cu-MMI NCs	8.5	100	-	-	8.5
Cu-MMI NCs/PVA solution	9.9	100	-	-	9.9
Au-ATT NCs	1.4	66	11.2	34	4.7
Au-ATT NCs/PVA solution	2.6	53	15.9	47	8.9
Au-BSA NCs	2.9	73	18	28	7.2
Au-BSA NCs/PVA solution	3.3	67	19	33	8.5

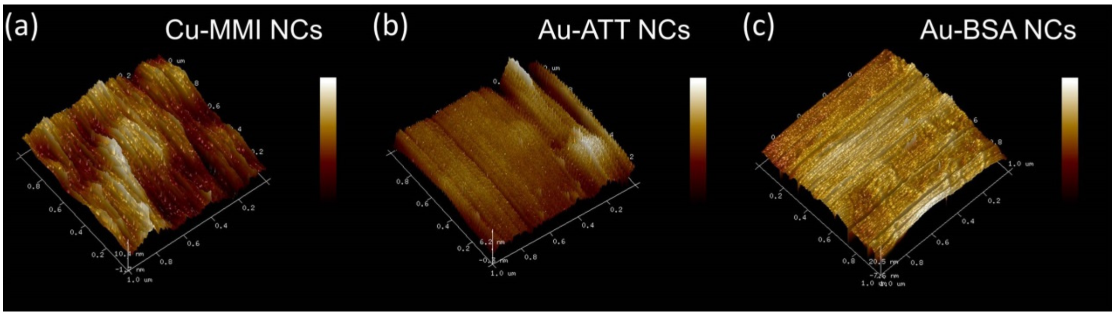

Figure S4. 3D AFM images of the (a) Cu-MMI NCs/PVA film, (b) Au-ATT NCs/PVA film and (c) Au-BSA NCs/PVA film.

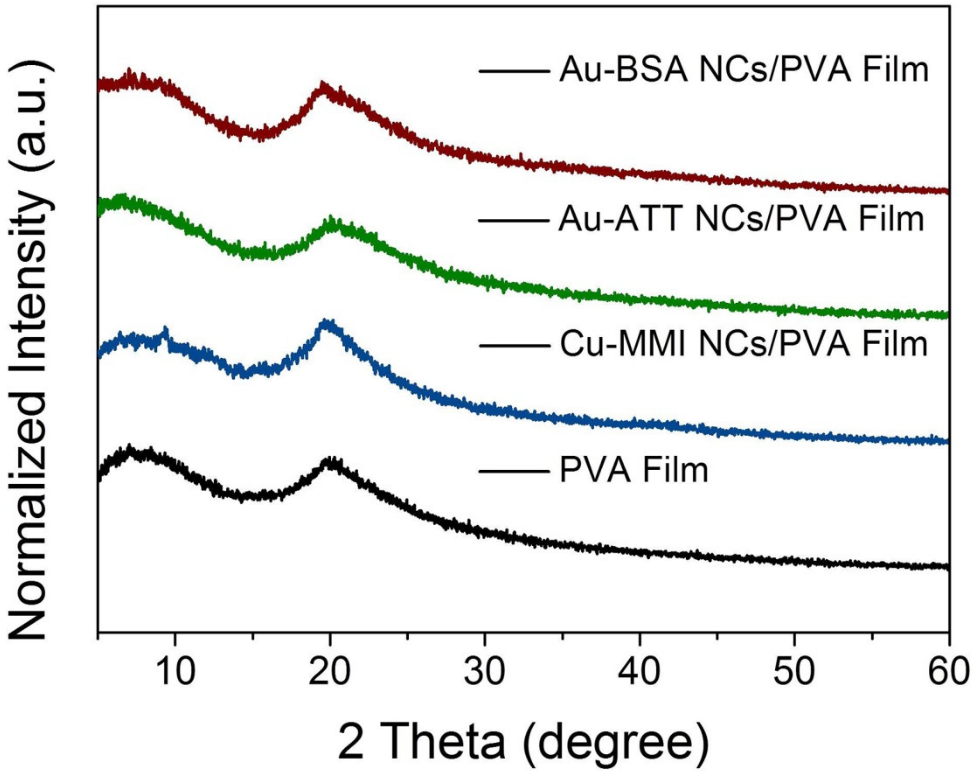

Figure S5. XRD patterns of PVA film, Cu-MMI NCs/PVA film, Au-ATT NCs/PVA film, and Au-BSA NCs/PVA film.

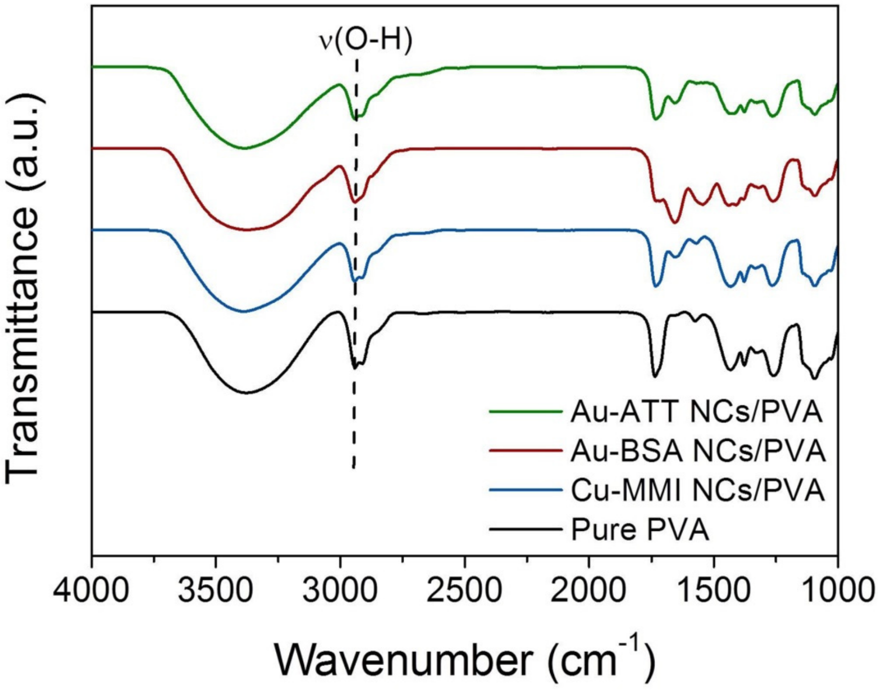

Figure S6. FTIR spectra of PVA, Cu-MMI NCs/PVA, Au-ATT NCs/PVA, and Au-BSA NCs/PVA composite films.

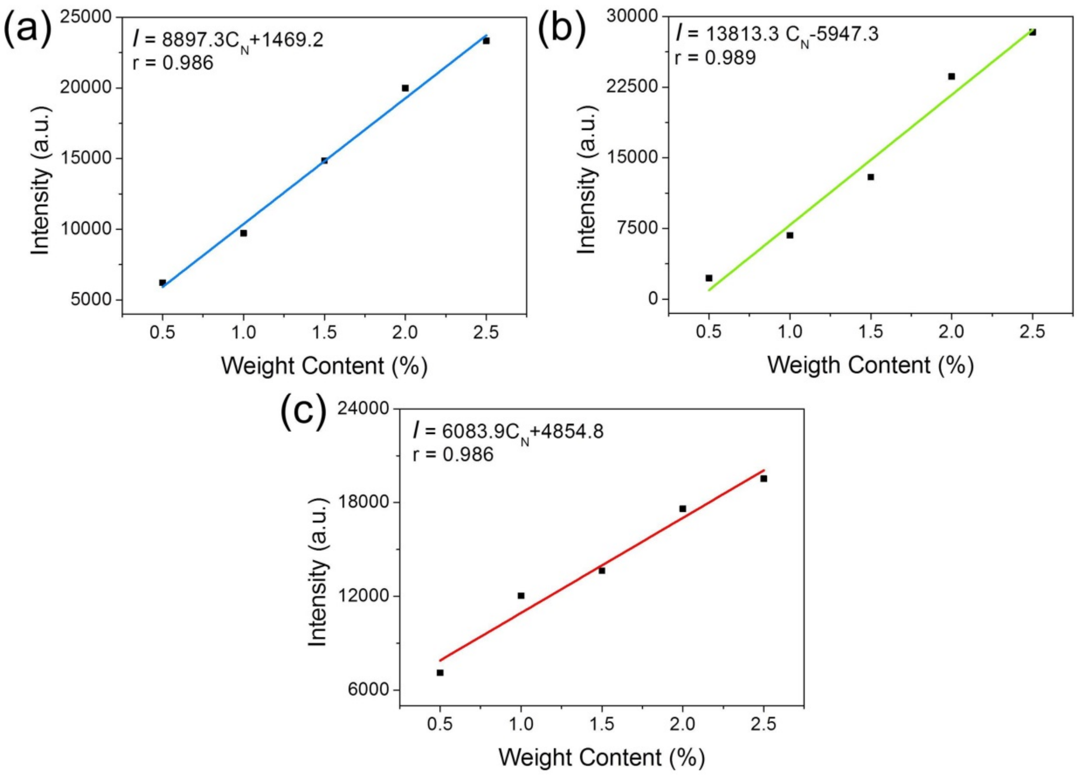

Figure S7. The linear regression curves of the PL emission intensity of (a) Cu-MMI NCs/PVA, (b) Au-ATT NCs/PVA, and (c) Au-BSA NCs/PVA composite films versus the weight content of MNCs.

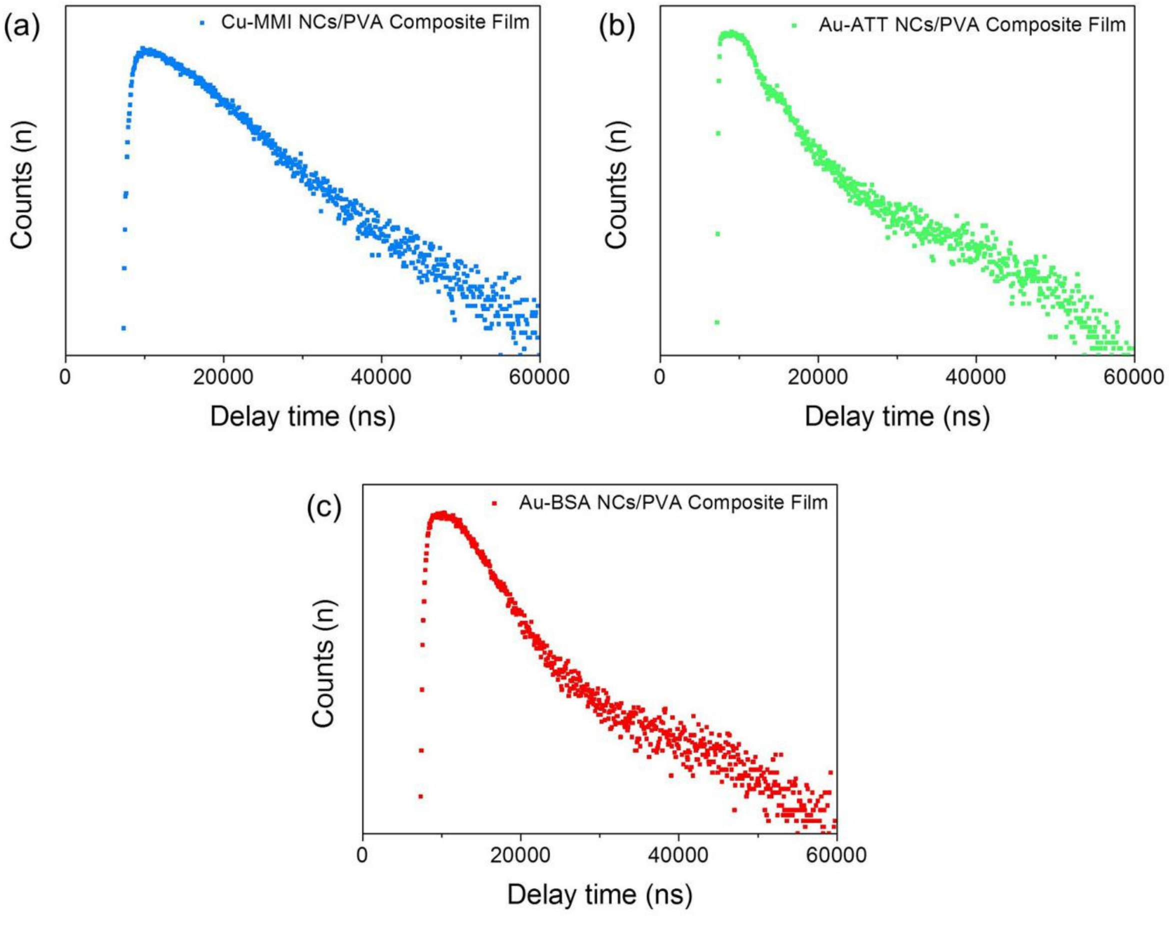

Figure S8. PL decay curves of the MNCs/PVA composite films, which are detected under 365 nm excitation. (a) Cu-MMI NCs, (b) Au-ATT NCs, and (c) Au-BSA NCs.

Table S3. Photophysical data of MNCs and MNCs/PVA composite films.

	$\tau_{average}$ (μs)	Φ_{PL} (%)	k_r (10^3 s^{-1})	k_{nr} (10^5 s^{-1})
Cu-MMI NCs	8.5	6.43	7.56	1.10
Cu-MMI NCs/PVA film	10.7	11.4	10.65	0.83
Au-ATT NCs	4.7	2.51	5.34	2.07
Au-ATT NCs/PVA film	11.2	10.4	9.29	0.80
Au-BSA NCs	7.2	5.25	7.29	1.32
Au-BSA NCs/PVA film	11.8	9.9	8.39	0.76

The rate constants of radiative (k_r) and nonradiative (k_{nr}) processes of the MNCs and MNCs/PVA films are calculated based on the Equation 1 and Equation 2, where Φ_{PL} is the photoluminescence quantum yield and $\tau_{average}$ is their average recombination lifetimes:

$$\Phi_{PL} = k_r \times \tau_{average} \qquad \text{Equation 1}$$

$$\tau_{average} = \frac{1}{(k_r + k_{nr})} \qquad \text{Equation 2}$$

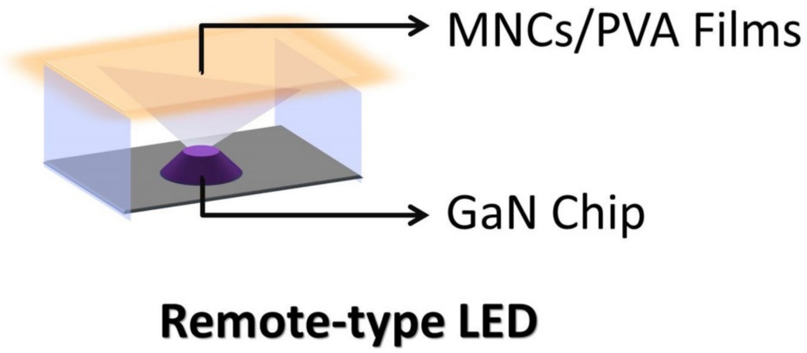

Figure S9. The structure illustration of a remote-type LED.

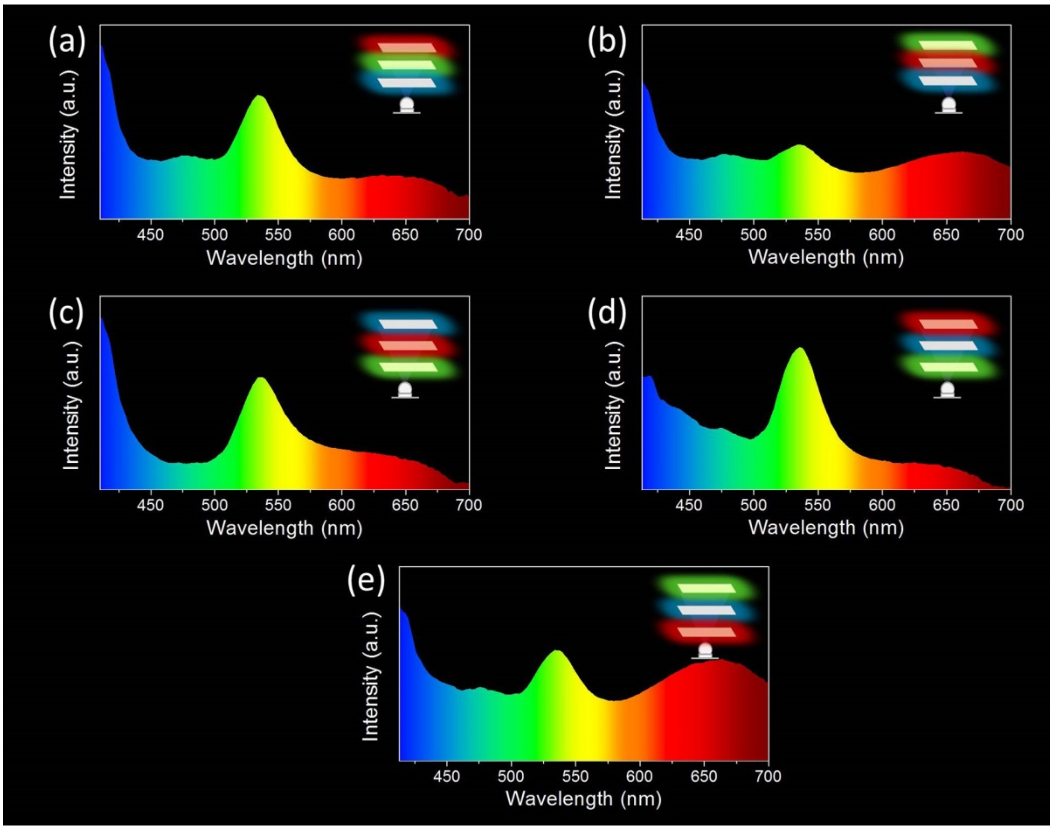

Figure S10. PL emission spectra of WLEDs based on the triple composite films with different arrangement of Cu-MMI NCs/PVA, Au-ATT NCs/PVA, and Au-BSA NCs/PVA films, where B represents blue, G represents green and R represents red. From bottom up: (a) B-G-R, (b) B-R-G, (c) G-R-B, (d) G-B-R and (e) R-B-G.

Table S4. Index parameters of WLEDs based on the triple composite films with different arrangement of Cu-MMI NCs/PVA, Au-ATT NCs/PVA, and Au-BSA NCs/PVA films from bottom up.

Sequence	Coordinate	CCT (K)	CRI	Luminance (cd/m^2)
B-G-R	(0.28,0.36)	7799	75	97.39
B-R-G	(0.31,0.32)	6704	84	82.06
G-R-B	(0.31,0.39)	6434	63	96.20
G-B-R	(0.25,0.36)	9490	58	89.65
R-B-G	(0.32,0.33)	5908	83	89.39
